# Supplementary material for: Damage Mechanisms in Polyalkenes Irradiated with Ultrashort XUV/X-Ray Laser Pulses
Source: J Phys Chem B. 2024 Sep 6;128(37):9036–42. doi: 10.1021/acs.jpcb.4c04126 (PMC11421090; doi:10.1021/acs.jpcb.4c04126)
Supplement: Supplementary file 1 — jp4c04126_si_001.pdf [file jp4c04126_si_001.pdf]

Supporting Information for Publication:

Damage mechanisms in polyalkenes irradiated with ultra-short XUV/x-ray laser pulses

Nikita Nikishev[1,2], Nikita Medvedev[1,3,*]

1) Institute of Physics, Czech Academy of Sciences, Na Slovance 1999/2, 182 00 Prague 8, Czech Republic

2) Czech Technical University in Prague, Faculty of Nuclear Sciences and Physical Engineering, Břehová 7, 115 19 Prague 1, Czech Republic

3) Institute of Plasma Physics, Czech Academy of Sciences, Za Slovankou 3, 182 00 Prague 8, Czech Republic

Atomic snapshots of polypropylene irradiated with the deposited dose of 0.05 eV/atom and 0.06 eV/atom are shown in Figure S1. The atoms in this Figure are color-coded according to their Mulliken charge [1]. It can be seen that the carbon with the broken bond acquires a local negative charge. In the example of 0.05 eV/atom, the carbon-carbon bond transiently breaks, forming a scission at the time of ~30-40 fs, which then recovers completely by the time of ~200 fs. This can also be seen in the transient formation and disappearance of the defect electronic energy levels shown in Figure S2 (cf. Figure 2 in the main text with the dose 0.06 eV/atom where stable defect levels are formed). In contrast, at the dose of 0.06 eV/atom, hydrogen dimer forms, also leaving a negatively charged carbon atom behind (Figure S1b).

[*] Corresponding author: nikita.medvedev@fzu.cz, Phone: +420 26605 2819

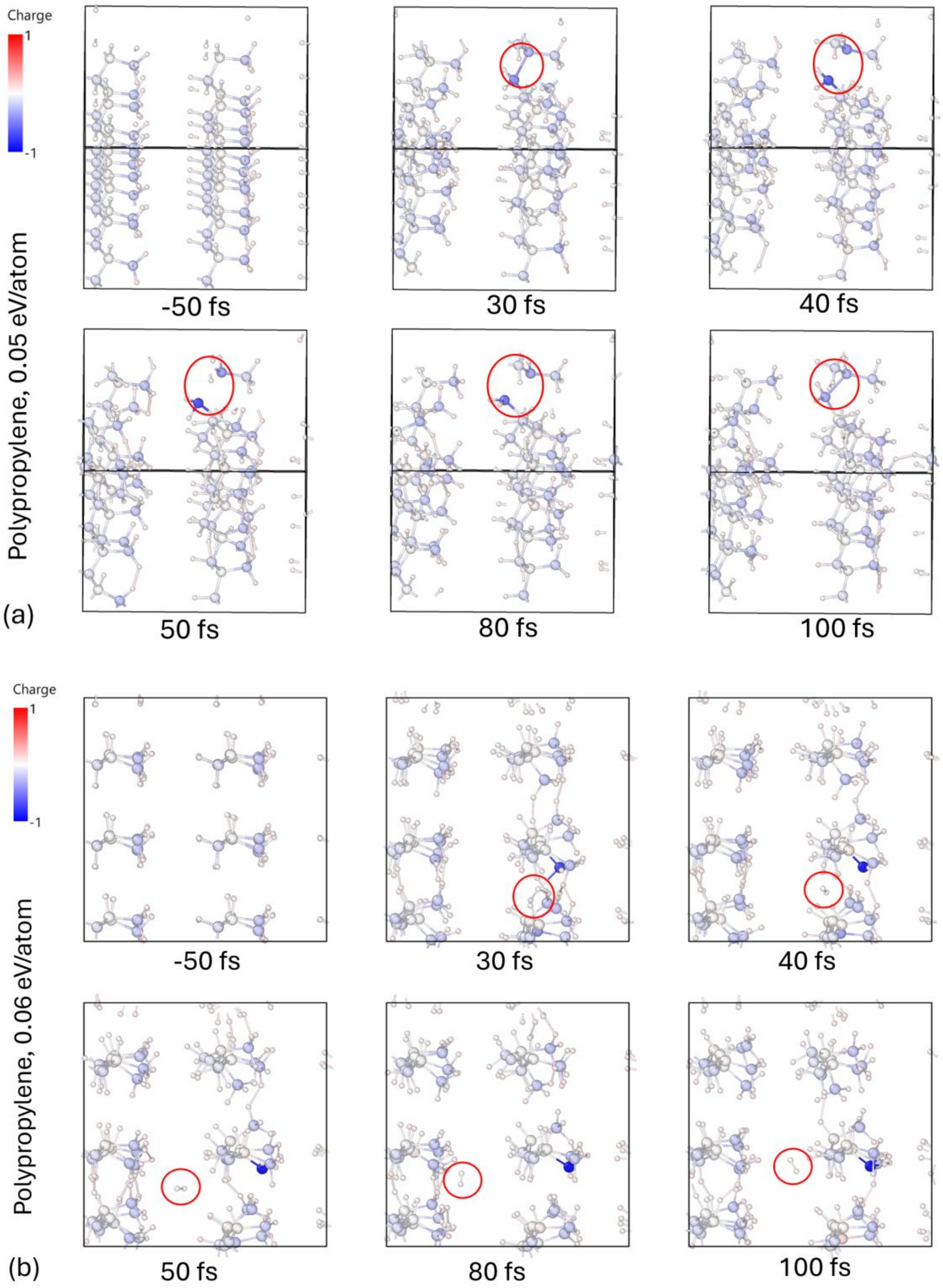

Figure S1. Atomic snapshots of polypropylene irradiated with 30 eV photons, 10 fs laser pulse with (a) 0.05 eV/atom, and (b) 0.06 eV/atom deposited dose. The atoms are color-coded corresponding to their Mulliken charge. The red circles highlight (a) transient chain scission (bond breaking) and recovery; and (b) dehydrogenation and emission of hydrogen dimer. Large balls are carbon atoms, small are hydrogen.

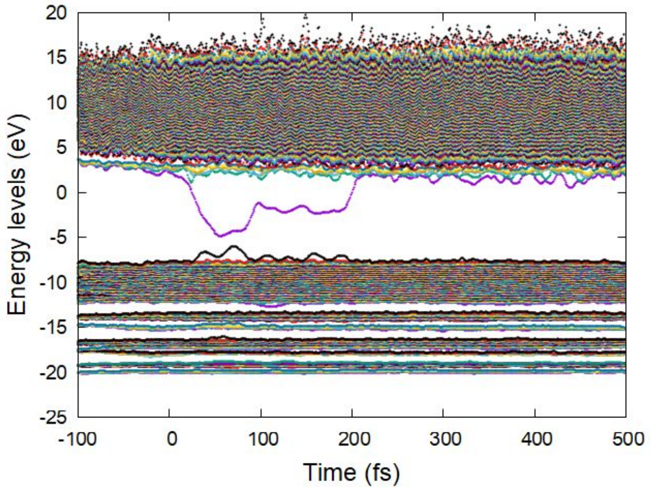

Figure S2. Electronic energy levels (molecular orbitals) in polypropylene irradiated with 30 eV photons, 10 fs FWHM FEL pulse, the absorbed dose of 0.05 eV/atom.

I. References

(1) Mulliken, R. S. Electronic Population Analysis on LCAO–MO Molecular Wave Functions. I. *J. Chem. Phys.* **1955**, *23* (10), 1833–1840. https://doi.org/10.1063/1.1740588.
